# Plasma p-tau217 best captures early longitudinal cognitive changes in subjective cognitive decline compared with p-tau181 and NfL

**DOI:** 10.1007/s00415-026-13966-z

**Published:** 2026-06-26

**Authors:** Giulia Giacomucci, Alice Pieri, Silvia Bagnoli, Katia Maria Giametta, Sonia Padiglioni, Valentina Moschini, Carmen Morinelli, Silvia Maria Rita Tabbì, Chiara Sensi, Lorenzo Mistretta, Chiara Crucitti, Benedetta Nacmias, Valentina Bessi

**Affiliations:** 1 Department of Neuroscience, Psychology, Drug Research and Child Health, University of Florence, Viale Pieraccini 6, 50134 Florence, Italy; 2 Dipartimento Neuroscienze E Degli Organi Di Senso, Neurology Unit, Careggi University Hospital, Florence, Italy; 3 Regional Referral Centre for Relational Criticalities - Tuscany Region, Florence, Italy; 4 IRCCS Fondazione Don Carlo Gnocchi, Florence, Italy

**Keywords:** Alzheimer's Disease, Subjective Cognitive Decline, Plasma p-tau217, NfL, Plasma p-tau181

## Abstract

**Background:**

Blood-based biomarkers are increasingly used to support the biological diagnosis of Alzheimer’s disease (AD), but their prognostic value in subjective cognitive decline (SCD) remains incompletely understood. We investigated whether plasma p-tau217, p-tau181, and neurofilament light chain (NfL) are associated with domain-specific longitudinal cognitive changes in SCD.

**Methods:**

76 SCD patients underwent plasma biomarker assessment and extensive neuropsychological evaluation at baseline and after two years. Participants were classified according to plasma biomarker status: p-tau217-negative, gray-zone, or positive; p-tau181-negative or positive; and NfL-negative or positive. Linear mixed effects models with random intercepts were used to assess the effects of time, biomarker status, and their interaction on cognitive outcomes.

**Results:**

Plasma p-tau217 showed the most consistent association with longitudinal cognitive trajectories. p-Tau217-positive participants declined in short story delayed recall and visual search, while gray-zone participants showed an intermediate memory trajectory. For category fluency, p-tau217-negative participants improved over time. Plasma p-tau181 showed weaker and less consistent associations, mainly involving memory measures and emerging primarily in post hoc analysis. NfL was associated with a more heterogeneous profile, without a clear pattern of longitudinal cognitive decline.

**Discussion:**

Plasma p-tau217 was associated with early domain-specific cognitive vulnerability in SCD, involving episodic memory and attentional functioning. These findings suggest that plasma p-tau217 showed more consistent associations with early domain-specific cognitive changes than p-tau181 and NfL, supporting its role in cognitive stratification of individuals within the earliest stages of the AD continuum.

**Supplementary Information:**

The online version contains supplementary material available at 10.1007/s00415-026-13966-z.

## Introduction

Plasma biomarkers have been recently validated for the biological diagnosis of Alzheimer’s disease (AD) [1], with the aim of making it more accessible. Indeed, identification of AD pathology previously required cerebrospinal fluid (CSF) biomarkers or amyloid positron emission tomography (amyloid-PET), which are more expensive and invasive tests [1, 2]. The role of amyloid beta (Aβ) 42, Aβ42/40 ratio, phosphorylated tau (p-tau217) and p-tau181 has been especially studied [3–6]. These biomarkers reflect brain Aβ burden: while Aβ indicates the presence of an amyloid beta proteinopathy [1, 3], p-tau217 and 181 reflect the pathophysiological connection between amyloid plaques’ accumulation and the resulting tau hyperphosphorylation and aggregration [7–9]. Moreover, other plasma biomarkers, such as neurofilament light chain (NfL) and glial fibrillar acidic protein (GFAP), although less specific for AD [1, 10], are useful biological indicators of neuroaxonal injury and astroglial activation respectively, and can have a prognostic value in AD [1, 10, 11]. Ultimately, plasma biomarkers can lead to a biological diagnosis in the early stages of the AD continuum, allowing to demonstrate an underlying AD pathology in preclinical stages such as Subjective Cognitive Decline (SCD) [1, 12, 13]. This can be especially helpful in a time where disease-modifying therapies are becoming increasingly available [1].

Moreover, plasma biomarkers have been associated with cognitive decline in the AD continuum, therefore proving to have not only a diagnostic role, but also prognostic value [14, 15]. Indeed, it has been demonstrated that levels of plasma biomarkers can predict the worsening of cognitive functions [14–16]. However, most studies considered global cognitive function, without evaluating specific cognitive domains [16], and those who did mostly explored such associations in AD dementia and Mild Cognitive Impairment (MCI), but not in SCD [16–20]. Finally, only a few studies aimed to investigate the association between plasma biomarkers and the change of performances in specific cognitive domains over time [16, 17].

In this work, we aimed to understand whether plasma biomarkers—notably p-tau217, p-tau181 and NfL—could have an influence on specific cognitive functions in SCD patients and, in particular, if there was an association between the status of plasma biomarkers and the change of neuropsychological tests’ scores over time.

## Materials and methods

### Participants

This study has been conducted as a part of the ongoing longitudinal study titled "Predicting the Evolution of Subjective Cognitive Decline to Alzheimer’s Disease With Machine Learning (PREVIEW)" (ClinicalTrials.gov Identifier: NCT05569083) [21]. We considered 76 SCD patients referred to the Center for Alzheimer’s Disease and Adult Cognitive Disorders of Careggi Hospital in Florence between January 2017 and March 2024. SCD was defined according to the criteria proposed by Jessen et al., requiring a self-experienced persistent decline in cognitive capacity compared with a previously normal status, not related to an acute event, in the absence of objective cognitive impairment [22]. All participants had normal age-, sex-, and education-adjusted performance on standardized neuropsychological tests and did not meet criteria for MCI or dementia. Normal cognitive performance was operationally defined as performance within 2 standard deviations from the normative mean in all administered cognitive tests. A score below this threshold in even one test was considered pathological and led to exclusion from the SCD group. All patients underwent blood collection for measurement of plasma biomarkers (p-tau217, p-tau181, NfL) (Table 1). All participants underwent a comprehensive family and clinical history, general and neurological examination, extensive neuropsychological investigation at baseline (T0) and after two years (T1), estimation of premorbid intelligence and assessment of depression at baseline. Positive family history was defined as one or more first-degree relatives with documented cognitive decline. Patients were classified categorized as positive or negative according to a previously defined single cut-off for p-tau181 (2.23 pg/ml) and for NfL (16.5389 pg/ml) [23]. As regards p-tau217, three groups of participants (that is, positive, negative and gray-zone) were defined using two different thresholds (0.214–0.391 pg/ml), as previously described [23, 24]. All subjects gave consent for *APOE* genotyping [25]. 60–80 patients also underwent FDG-PET scans. The local ethics committee approved the protocol of the study. All participants gave written informed consent to participate in the study.

**Table 1:** Demographic, clinical, and biological features of the SCD cohort

	SCD
Age at onset	57.47 ± 9.03
Age at baseline	60.80 ± 8.78
Age at plasma collection	64.64 ± 8.29
Years of education	13.21 ± 8.78
Sex (M – F)	18—58
Family history	72.36%
*APOE* ε4 status	28.57%
Plasma p-tau217	
Positive	19.30%
Gray Zone	31.58%
Negative	49.12%
Plasma p-tau181	
Positive	38.60%
Negative	61.40%
Plasma NfL	
Positive	22.54%
Negative	77.46%

Values are reported as mean ± standard deviation for continuous variables and as percentages for categorical variables, unless otherwise specified

*SCD* subjective cognitive decline; *APOE* apolipoprotein E; p-tau phosphorylated tau; NfL neurofilament light chain

### Neuropsychological evaluation

All participants underwent a comprehensive neuropsychological assessment covering global cognition, memory, attention, language, visuoconstructive abilities, and executive functioning. Global cognitive status was assessed with the Mini-Mental State Examination (MMSE). Verbal and visuospatial short-term and working memory were evaluated using forward and backward Digit Span and Visuo-spatial Span tasks [26]. Long-term memory was examined through the Rey Auditory Verbal Learning Test, including immediate and delayed recall [27], the Babcock Short Story immediate and delayed recall [28], and the delayed recall of the Rey–Osterrieth Complex Figure [29]. Attentional abilities were assessed with the Trail Making Test A [30] and a visual search task [31]. Language was investigated using category fluency [32] and phonemic fluency tasks [33], together with the Screening for Aphasia in NeuroDegeneration battery [34]. Visuoconstructive abilities were assessed by the copy condition of the Rey–Osterrieth Complex Figure [35], while executive functioning was explored using the Trail Making Test B [30] and the Stroop Test [36]. Premorbid intellectual level was estimated at baseline with the Test di Intelligenza Breve (TIB) [37], the Italian adaptation of the National Adult Reading Test (NART) [38]. The severity of subjective memory complaints was assessed using the Memory Assessment Clinics-Questionnaire (MAC-Q) [39].

### Plasma collection and biomarkers analysis

Venous blood was drawn at the Neurology Unit of Careggi University Hospital using standard polypropylene EDTA tubes (Sarstedt, Nümbrecht, Germany). Samples were processed within two hours of collection by centrifugation at 1300 rcf for 10 min at room temperature. The plasma fraction was then separated and stored at − 80 °C until biomarker analysis, which was performed at the Laboratory of Neurogenetics of Careggi University Hospital. Plasma p-tau217 concentrations were quantified using a fully automated chemiluminescent enzyme immunoassay on the LUMIPULSE G600II platform, following the manufacturer’s protocol (Lumipulse^®^ assay, Fujirebio; research use only). Plasma NfL and p-tau181 were measured using single-molecule array technology on the Simoa SR-X platform, with reagents supplied by Quanterix Corporation (Lexington, MA, USA), according to the manufacturer’s instructions. NfL was analyzed using the Simoa NF-Light SR-X kit (cat. No. 103400). The lower limit of quantification and limit of detection were 0.316 pg/mL and 0.0552 pg/mL, respectively. All NfL measurements were performed in a single analytical run. Low- and high-concentration quality controls, corresponding to 5.08 pg/mL and 169 pg/mL, were included in the assay. The obtained values were within the expected range, with coefficients of variation below 20%. Plasma p-tau181 was quantified using the Simoa Human p-tau181 Advantage V2 kit (item No. 103714; Quanterix, Billerica, MA, USA). The analytical lower limit of quantification was 0.085 pg/mL, and the limit of detection was 0.041 pg/mL. Each run included seven calibrators and two quality controls provided by the manufacturer. Calibrators were used to generate the standard curve, while the controls represented low and high target concentrations. Plasma samples and controls were diluted four-fold and analyzed in duplicate [23, 40, 41].

### Statistical analysis

Statistical analysis was performed using R software, version 4.5.1 (R Foundation for Statistical Computing, Vienna, 2013). Normality of variable distributions was assessed with the Shapiro–Wilk test. Continuous data were described using means and standard deviations, while categorical data were summarized as frequencies, percentages, and, where applicable, 95% confidence intervals. Group comparisons were performed using Mann–Whitney U tests for continuous variables and chi-square tests for categorical variables. Depending on the analysis, effect sizes were expressed as partial η^2^, Cohen’s d, rank-biserial correlation coefficient, or Cramer’s V. Longitudinal associations between plasma biomarker status and cognitive performance were examined using linear mixed effects models including random intercepts for participants. Fixed effects included time, biomarker status, and their interaction. For p-tau181 and NfL, biomarker status was classified as negative or positive, whereas p-tau217 status was classified as negative, gray-zone, or positive. Statistical tests were two-tailed, with significance set at *p* < 0.05.

## Results

### Longitudinal neuropsychological changes according to p-tau217 status

**Fig. 1:**
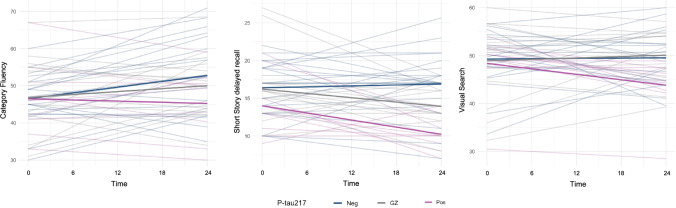
Longitudinal cognitive trajectories according to plasma p-tau217 status in patients with Subjective Cognitive Decline. Individual and group-level longitudinal trajectories of short story delayed recall, visual search, and category fluency over 24 months according to plasma p-tau217 status. Participants were classified as p-tau217-negative, gray-zone, or positive. Thin lines represent individual trajectories, while thick lines represent group-level trends. *GZ* gray-zone; *Neg* negative; *Pos* positive; *p-tau* phosphorylated tau

In episodic memory, short story delayed recall showed no main effect of time or p-tau217 status. However, time × p-tau217 interaction was represented by two model terms, with the p-tau217-negative group as the reference category. Compared with the negative group, the p-tau217-positive group showed a significantly different longitudinal trajectory (β − 4.28, SE 1.63, *p* = 0.012), while the gray-zone group showed a trend toward a different trajectory (β − 2.79, SE 1.41, *p* = 0.055). Post hoc analysis showed stability in the negative group (Δ − 0.51, *p* = 0.57) and decline in both the gray-zone (Δ − 2.29, *p* = 0.043) and positive groups (Δ − 3.78, *p* = 0.009). A similar pattern was observed for visual search, with no main effects of time or p-tau217 status. Considering time x p-tau217 interaction, compared with the p-tau217-negative group, the p-tau217-positive group showed a significantly different longitudinal trajectory (β − 4.95, SE 2.42, *p* = 0.047). Post hoc analysis confirmed decline only in the positive group (Δ − 4.57, *p* = 0.024). For category fluency, a main effect of time was observed (β 6.02, SE 1.75, *p* = 0.001), with no main effect of p-tau217 status, but a significant time × p-tau217 interaction, with for the positive group showing a different longitudinal trajectory compared with negative-one (β − 7.31, SE 3.12, *p* = 0.024). Post hoc analysis showed improvement in the negative group (Δ 6.02, *p* = 0.001), a mild not-significant improvement in the gray-zone group (Δ 3.16, *p* = 0.139), and a slight but not significant decline in the positive group (Δ − 1.29, *p* = 0.622). By contrast, p-tau217 was not associated with differential longitudinal change in other attentive/executive measures. For TMT A, a main effect of time was found (β 6.48, SE 2.94, *p* = 0.034), without effects of p-tau217 status or interaction: post hoc analysis showed a significant increase in completion time only in the negative group (Δ 6.48, *p* = 0.034). Similarly, TMT B showed a main effect of time (β 22.38, SE 6.44, *p* = 0.001), with no main or interaction effects of p-tau217 status, showing significant worsening in the negative group (Δ 22.40, *p* = 0.001). Finally, for phonemic fluency, time was again significant (β 5.06, SE 1.94, *p* = 0.012), but no p-tau217 or interaction effects were detected, with a significant improvement only in the negative group (Δ 5.06, *p* = 0.012) (Fig. 1; Table 2; Supplementary Table 1).

**Table 2:** Estimated marginal means and longitudinal within-group changes in neuropsychological performance according to plasma p-tau217 status

	p-tau217 negative			p-tau217 gray zone			p-tau217 positive		
	Emmeans ± SE	T	*p*	Emmeans ± SE	T	*p*	Emmeans ± SE	T	*p*
MMSE T0	28.30 ± 0.37	−1.21	0.230	28.60 ± 0.45	0.17	0.860	27.30 ± 0.53	−1.08	0.282
MMSE T1	28.80 ± 0.37			28.50 ± 0.45			28.00 ± 0.56		
Short Story IR T0	12.00 ± 0.84	−0.76	0.451	13.00 ± 1.04	0.93	0.357	10.67 ± 1.30	1.45	0.154
Short Story IR T1	12.76 ± 0.84			11.86 ± 1.04			8.44 ± 1.30		
Short Story DR T0	16.40 ± 0.84	−0.56	0.573	16.20 ± 1.04	2.08	**0.043**	14.00 ± 1.30	2.76	**0.008**
Short Story DR T1	16.90 ± 0.84			13.90 ± 1.04			10.20 ± 1.30		
RAVLT IR T0	48.50 ± 2.10	1.46	0.150	47.90 ± 2.57	1.77	0.083	43.50 ± 3.15	0.62	0.533
RAVLT IR T1	45.80 ± 2.10			43.90 ± 2.57			41.60 ± 3.15		
RAVLT DR T0	10.96 ± 0.59	1.65	0.106	10.71 ± 0.73	1.64	0.108	9.87 ± 0.90	1.65	0.106
RAVLT DR T1	9.98 ± 0.59			9.51 ± 0.73			8.32 ± 0.90		
Rey Figure copy T0	34.90 ± 0.64	0.55	0.581	33.50 ± 0.79	0.71	0.480	32.00 ± 0.93	−0.75	0.454
Rey Figure Copy T1	34.40 ± 0.66			32.70 ± 0.82			33.00 ± 0.99		
Rey Figure recall T0	22.30 ± 1.18	−0.44	0.655	19.20 ± 1.45	0.12	0.905	20.60 ± 1.71	1.45	0.154
Rey Figure recall T1	22.80 ± 1.18			19.00 ± 1.45			18.20 ± 1.78		
Category Fluency T0	46.70 ± 2.05	−3.44	**0.003**	46.80 ± 2.47	−1.51	0.138	46.50 ± 2.93	0.49	0.621
Category Fluency T1	52.70 ± 2.02			50.00 ± 2.47			45.20 ± 3.02		
Phonemic Fluency T0	37.70 ± 2.56	−2.60	**0.012**	37.70 ± 3.13	−1.42	0.162	35.80 ± 3.71	−0.44	0.660
Phonemic Fluency T1	42.80 ± 2.56			41.10 ± 3.13			37.10 ± 3.81		
Stroop Test T0	13.90 ± 2.06	−1.24	0.291	14.60 ± 2.50	0.15	0.880	20.40 ± 2.95	0.69	0.488
Stroop Test T1	15.50 ± 2.04			14.40 ± 2.50			18.90 ± 3.09		
TMT A T0	28.90 ± 3.38	−2.20	**0.033**	28.80 ± 4.24	−0.29	0.770	34.60 ± 5.27	−0.51	0.607
TMT A T1	35.30 ± 3.38			29.90 ± 4.14			37.10 ± 5.07		
TMT B T0	60.10 ± 7.97	−3.47	**0.001**	52.10 ± 9.97	−1.89	0.065	74.80 ± 12.30	−1.68	0.099
TMT B T1	82.50 ± 7.97			67.60 ± 9.77			93.00 ± 11.90		
Visual Search T0	49.30 ± 1.39	−0.26	0.789	48.90 ± 1.67	−0.71	0.480	48.40 ± 1.98	2.336	**0.024**
Visual Search T1	49.70 ± 1.42			50.10 ± 1.67			43.80 ± 1.98		
FAB T0	16.60 ± 0.34	−0.80	0.426	16.70 ± 0.42	0.43	0.669	15.80 ± 0.49	0.88	0.379
FAB T1	16.90 ± 0.35			16.40 ± 0.43			15.30 ± 0.49		
Naming T0	13.90 ± 0.17	1.02	0.313	13.60 ± 0.22	−0.28	0.779	13.70 ± 0.26	1.08	0.283
Naming T1	13.70 ± 0.16			13.70 ± 0.19			13.30 ± 0.23		
Verbal Span forward T0	5.92 ± 0.21	0.51	0.614	6.54 ± 0.26	0.51	0.612	5.73 ± 0.32	−0.85	0.398
Verbal Span forward T1	5.79 ± 0.21			6.38 ± 0.26			6.06 ± 0.34		
Verbal Span backward T0	4.40 ± 0.24	1.45	0.153	4.79 ± 0.29	0.03	0.975	4.37 ± 0.37	1.01	0.317
Verbal Span backward T1	4.04 ± 0.24			4.78 ± 0.29			3.97 ± 0.38		
Spatial Span forward T0	5.27 ± 0.17	1.58	0.120	5.28 ± 0.22	1.74	0.08	4.70 ± 0.26	0.50	0.615
Spatial Span forward T1	4.92 ± 0.17			4.80 ± 0.21			4.53 ± 0.26		
Spatial Span backward T0	4.60 ± 0.21	−0.53	0.598	4.85 ± 0.26	0.84	0.405	4.52 ± 0.32	1.00	0.321
Spatial Span backward T1	4.74 ± 0.21			4.58 ± 0.25			4.13 ± 0.32		

Values represent estimated marginal means (emmeans ± standard error) derived from linear mixed effects models with random intercepts for participants. For each cognitive outcome, time (T0 vs T1), plasma p-tau217 status (negative, gray-zone, positive), and their interaction were included as predictors. T and p values refer to within-group comparisons over time (T0 vs T1) based on model-derived contrasts. Positive T values indicate an increase over time, whereas negative T values indicate a decrease. For timed measures (e.g., Trail Making Test, Stroop), higher scores reflect worse performance.

*IR* immediate recall; *DR* delayed recall; *RAVLT* Rey Auditory Verbal Learning Test; *FAB* Frontal Assessment Battery

Statistically significant results are highlighted in **bold**

### Longitudinal neuropsychological changes according to p-tau181 status

**Fig. 2:**
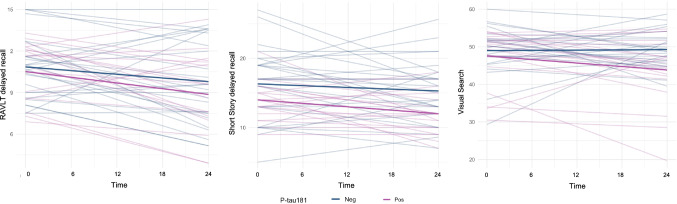
Longitudinal cognitive trajectories according to plasma p-tau181 status in patients with Subjective Cognitive Decline. Individual and group-level longitudinal trajectories of short story delayed recall, visual search, and Rey Auditory Verbal Learning Test delayed recall over 24 months according to plasma p-tau181 status. Participants were classified as p-tau181 negative or positive. Thin lines represent individual trajectories, while thick lines represent group-level trends. Neg = negative; Pos = positive; p-tau = phosphorylated tau

For episodic memory, short story delayed recall showed no main effect of time and no interaction, with a trend toward a main effect of p-tau181 (β − 2.31, SE 1.31, *p* = 0.082). Post hoc analysis showed decline only in the positive group (Δ − 2.00, *p* = 0.048). Similarly, RAVLT immediate recall showed no main or interaction effects although post hoc analysis indicated decline in the positive group (Δ − 5.04, *p* = 0.012). For RAVLT delayed recall, a main effect of time was observed (β − 1.07, SE 0.50, *p* = 0.040), without main or interaction effects of p-tau181; post hoc analysis showed decline in both groups, greater in the positive group (negative: Δ − 1.07, *p* = 0.040; positive: Δ − 1.63, *p* = 0.009). For visual search, no main effects of time and p-tau181 status were detected, but a trend toward interaction emerged (β − 3.86, SE 2.23, *p* = 0.090); post hoc analysis showed decline only in the positive group (Δ − 3.56, *p* = 0.039). For TMT B, both time (β 24.15, SE 6.79, *p* = 0.001) and p-tau181 status (β 24.06, SE 11.26, *p* = 0.036) were significant, whereas the interaction was not, with post hoc analysis showing a significant increase in execution time in the negative group (Δ 24.10, *p* = 0.001). For category and phonemic fluency, only time was significant (category fluency: β 4.90, SE 1.56, *p* = 0.003; phonemic fluency: β 4.593, SE 1.784, *p* = 0.013), with improvement limited to the negative group (category fluency: Δ 4.90, *p* = 0.003, phonemic fluency: Δ 5.10, *p* = 0.012). Overall, p-tau181 showed a weaker and less consistent pattern than p-tau217, with effects mainly emerging in memory measures and at the post hoc level (Fig. 2; Table 3; Supplementary Table 2).

**Table 3:** Estimated marginal means and longitudinal within-group changes in neuropsychological performance according to plasma p-tau181 status

	p-tau181 negative			p-tau181 positive		
	Emmeans ± SE	T	*p*	Emmeans ± SE	T	*P*
MMSE T0	27.90 ± 0.34	−2.00	0.051	28.20 ± 0.39	0.64	0.522
MMSE T1	28.60 ± 0.34			27.90 ± 0.40		
Short Story IR T0	12.23 ± 0.81	0.35	0.722	11.11 ± 0.98	1.45	0.154
Short Story IR T1	11.92 ± 0.81			9.61 ± 0.98		
Short Story DR T0	16.30 ± 0.83	1.25	0.216	14.00 ± 1.00	2.03	**0.048**
Short Story DR T1	15.30 ± 0.83			12.00 ± 1.00		
RAVLT IR T0	48.00 ± 1.87	1.47	0.147	46.90 ± 2.17	2.62	**0.012**
RAVLT IR T1	45.60 ± 1.87			41.80 ± 2.17		
RAVLT DR T0	10.85 ± 0.52	2.11	**0.040**	10.42 ± 0.60	2.72	**0.009**
RAVLT DR T1	9.79 ± 0.52			8.79 ± 0.60		
Rey Figure copy T0	34.30 ± 0.65	0.55	0.585	33.10 ± 0.76	1.32	0.194
Rey Figure Copy T1	33.80 ± 0.66			31.70 ± 0.78		
Rey Figure recall T0	21.20 ± 1.14	0.17	0.865	20.10 ± 1.30	1.32	0.191
Rey Figure recall T1	21.00 ± 1.14			18.50 ± 1.32		
Category Fluency T0	46.80 ± 1.91	−3.13	**0.003**	47.70 ± 2.18	−1.30	0.200
Category Fluency T1	51.70 ± 1.89			50.10 ± 2.18		
Phonemic Fluency T0	38.60 ± 2.23	−2.57	**0.013**	37.50 ± 2.54	−0.94	0.350
Phonemic Fluency T1	43.20 ± 2.23			39.50 ± 2.58		
Stroop Test T0	14.80 ± 2.02	0.15	0.882	16.00 ± 2.34	−1.76	0.085
Stroop Test T1	14.50 ± 2.02			19.90 ± 2.43		
TMT A T0	25.60 ± 2.88	−1.86	0.069	29.70 ± 3.37	−1.04	0.300
TMT A T1	31.10 ± 2.83			33.40 ± 3.29		
TMT B T0	51.50 ± 7.33	−3.55	**0.001**	75.60 ± 8.55	−1.95	0.057
TMT B T1	75.70 ± 7.23			91.80 ± 8.55		
Visual Search T0	49.00 ± 1.38	−0.19	0.842	47.50 ± 1.55	2.13	**0.038**
Visual Search T1	49.30 ± 1.38			44.00 ± 1.58		
FAB T0	16.70 ± 0.32	−0.42	0.669	16.20 ± 0.36	1.14	0.258
FAB T1	16.80 ± 0.32			15.70 ± 0.37		
Naming T0	13.90 ± 0.15	0.25	0.803	13.70 ± 0.17	1.48	0.145
Naming T1	13.80 ± 0.14			13.40 ± 0.16		
Verbal Span forward T0	6.190 ± 0.19	1.08	0.285	5.95 ± 0.23	0.34	0.733
Verbal Span forward T1	5.950 ± 0.19			5.86 ± 0.23		
Verbal Span backward T0	4.50 ± 0.20	1.75	0.086	4.03 ± 0.23	−0.32	0.748
Verbal Span backward T1	4.12 ± 0.19			4.12 ± 0.24		
Spatial Span forward T0	5.07 ± 0.17	1.05	0.298	5.20 ± 0.20	1.86	0.069
Spatial Span forward T1	4.86 ± 0.16			4.78 ± 0.20		
Spatial Span backward T0	4.67 ± 0.19	0.06	0.952	4.77 ± 0.22	1.29	0.202
Spatial Span backward T1	4.66 ± 0.18			4.43 ± 0.22		

Values represent estimated marginal means (emmeans ± standard error) derived from linear mixed effects models with random intercepts for participants. For each cognitive outcome, time (T0 vs T1), plasma p-tau181 status (negative, positive), and their interaction were included as predictors. T and p values refer to within-group comparisons over time (T0 vs T1) based on model-derived contrasts. Positive T values indicate an increase over time, whereas negative T values indicate a decrease. For timed measures (e.g., Trail Making Test, Stroop), higher scores reflect worse performance.

*IR* immediate recall; *DR* delayed recall; *RAVLT* Rey Auditory Verbal Learning Test; *FAB* Frontal Assessment Battery

Statistically significant results are highlighted in **bold**

### Longitudinal neuropsychological changes according to NfL status

**Fig. 3:**
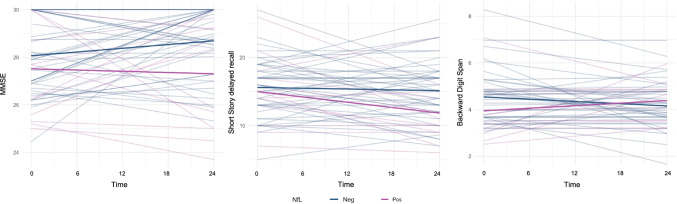
Longitudinal cognitive trajectories according to plasma NfL status in patients with Subjective Cognitive Decline. Individual and group-level longitudinal trajectories of short story delayed recall, backward digit span, and Mini-Mental State Examination over 24 months according to plasma NfL status. Participants were classified as NfL-negative or NfL-positive. Thin lines represent individual trajectories, while thick lines represent group-level trends. *Neg* negative; *Pos* positive; *NfL* neurofilament light chain

For global cognition (MMSE), a main effect of time was observed (β 0.65, SE 0.30, *p* = 0.034), without effects of NfL status or interaction; post hoc analysis showed a very mild improvement only in the NfL-negative group (Δ 0.65, *p* = 0.034). For short story delayed recall, no main effects were detected, but a trend toward interaction was observed (β − 2.69, SE 1.41, *p* = 0.063), with decline only in the NfL-positive group on post hoc testing (Δ − 3.17, *p* = 0.014). For RAVLT immediate and delayed recall, time effects were observed (immediate recall: β − 3.48, SE 1.46, *p* = 0.023; delayed recall: β − 1.02, SE 0.43, *p* = 0.022), without NfL main or interaction effects. Post hoc analysis demonstrated a significant decline in the NfL-negative group for both immediate (Δ − 3.48, *p* = 0.023) and delayed recall (Δ − 1.02, *p* = 0.022), and a trend non-significant decline in the NfL-positive group. For TMT A and TMT B, significant time effects were also found (TMT A: β 5.48, SE 2.48, *p* = 0.032; TMT B: β 19.01, SE 5.10, *p* < 0.001), with a significant decline in the negative group (TMT A: Δ − 2.20, *p* = 0.031; TMT B: Δ − 3.48, *p* = 0.023). Similarly, phonemic and category fluency likewise showed time effects (phonemic: β 3.94, SE 1.29, *p* = 0.004; category: β 3.18, SE 1.39, *p* = 0.026), but no main or interaction effects of NfL. A different pattern emerged for working memory. In backward digit span, both time (β − 0.38, SE 0.17, *p* = 0.027) and the time × NfL interaction (β 0.82, SE 0.35, *p* = 0.022) were significant, with decline in the NfL-negative group (Δ − 0.38, *p* = 0.026) and no significant change in the positive group. In forward spatial span, both time (β − 0.43, SE 0.15, *p* = 0.005) and NfL status (β − 0.59, SE 0.28, *p* = 0.034) were significant, whereas the interaction was not, indicating lower overall performance in NfL-positive participants but no differential longitudinal trajectory (Fig. 3; Table 4; Supplementary Table 3).

**Table 4:** Estimated marginal means and longitudinal within-group changes in neuropsychological performance according to plasma NfL status

	NfL-negative			NfL-positive		
	Emmeans ± SE	T	*P*	Emmeans ± SE	T	*p*
MMSE T0	28.10 ± 0.26	−2.17	**0.034**	27.50 ± 0.47	0.39	0.695
MMSE T1	28.70 ± 0.26			27.30 ± 0.47		
Short Story IR T0	11.78 ± 0.65	−0.03	0.973	11.17 ± 1.22	0.91	0.364
Short Story IR T1	11.80 ± 0.65			9.92 ± 1.22		
Short Story DR T0	15.60 ± 0.70	0.71	0.477	15.00 ± 1.30	2.54	**0.013**
Short Story DR T1	15.10 ± 0.70			11.80 ± 1.30		
RAVLT IR T0	47.70 ± 1.46	2.11	**0.039**	46.10 ± 2.69	1.68	0.097
RAVLT IR T1	44.90 ± 1.47			42.00 ± 2.62		
RAVLT DR T0	10.63 ± 0.41	2.36	**0.021**	9.96 ± 0.77	1.71	0.092
RAVLT DR T1	9.60 ± 0.42			8.60 ± 0.74		
Rey Figure copy T0	34.00 ± 0.49	0.59	0.558	32.70 ± 0.90	0.48	0.631
Rey Figure Copy T1	33.60 ± 0.50			32.10 ± 0.90		
Rey Figure recall T0	20.90 ± 0.86	−0.001	0.999	19.80 ± 1.55	0.22	0.822
Rey Figure recall T1	20.90 ± 0.86			19.50 ± 1.55		
Category Fluency T0	47.70 ± 1.44	−2.28	**0.026**	43.50 ± 2.65	−0.88	0.378
Category Fluency T1	50.90 ± 1.44			45.70 ± 2.57		
Phonemic Fluency T0	37.70 ± 1.75	−3.05	**0.003**	37.50 ± 2.54	−0.97	0.333
Phonemic Fluency T1	41.60 ± 1.76			39.50 ± 2.58		
Stroop Test T0	14.70 ± 1.47	−0.48	0.631	17.60 ± 2.72	−1.11	0.271
Stroop Test T1	15.30 ± 1.51			20.20 ± 2.65		
TMT A T0	27.20 ± 2.55	−2.20	**0.031**	33.80 ± 4.83	−0.56	0.576
TMT A T1	32.70 ± 2.55			36.40 ± 4.54		
TMT B T0	59.30 ± 5.81	−3.72	** < 0.001**	73.90 ± 10.90	−0.74	0.463
TMT B T1	78.30 ± 5.81			81.30 ± 10.60		
Visual Search T0	48.50 ± 1.10	−0.40	0.686	46.80 ± 1.97	1.75	0.085
Visual Search T1	48.90 ± 1.11			43.40 ± 1.97		
FAB T0	16.60 ± 0.25	−0.11	0.912	16.00 ± 0.44	0.53	0.596
FAB T1	16.60 ± 0.25			15.70 ± 0.44		
Naming T0	13.80 ± 0.11	1.04	0.302	13.60 ± 0.21	0.41	0.685
Naming T1	13.70 ± 0.10			13.50 ± 0.19		
Verbal Span forward T0	6.19 ± 0.15	1.62	0.111	5.82 ± 0.27	−1.50	0.138
Verbal Span forward T1	5.93 ± 0.15			6.27 ± 0.27		
Verbal Span backward T0	4.52 ± 0.17	2.28	**0.026**	3.95 ± 0.31	−1.44	0.154
Verbal Span backward T1	4.14 ± 0.17			4.39 ± 0.31		
Spatial Span forward T0	5.30 ± 0.13	2.92	**0.005**	4.70 ± 0.24	0.48	0.631
Spatial Span forward T1	4.87 ± 0.13			4.57 ± 0.24		
Spatial Span backward T0	4.74 ± 0.14	0.87	0.388	4.39 ± 0.26	0.04	0.967
Spatial Span backward T1	4.58 ± 0.14			4.38 ± 0.26		

Values represent estimated marginal means (emmeans ± standard error) derived from linear mixed effects models with random intercepts for participants. For each cognitive outcome, time (T0 vs T1), plasma NfL status (negative, positive), and their interaction were included as predictors. T and p values refer to within-group comparisons over time (T0 vs T1) based on model-derived contrasts. Positive T values indicate an increase over time, whereas negative T values indicate a decrease. For timed measures (e.g., Trail Making Test, Stroop), higher scores reflect worse performance.

*IR* immediate recall; *DR* delayed recall; *RAVLT* Rey Auditory Verbal Learning Test; *FAB* Frontal Assessment Battery

Statistically significant results are highlighted in **bold**

## Discussion

In this longitudinal study on SCD, we found that plasma biomarkers were associated with distinct cognitive trajectories over a two-year follow-up. In particular, plasma p-tau217 showed the most consistent relationship with longitudinal cognitive change**,** particularly in episodic memory and attentional functioning. By contrast, p-tau181 showed a weaker and less consistent pattern, mainly involving memory measures, whereas NfL was associated with a more heterogeneous profile, in keeping with its role as a non-specific marker of neuroaxonal injury. Taken together, these findings suggest that plasma p-tau217 may capture early AD-related cognitive vulnerability already at the SCD stage, before the emergence of objective clinical impairment.

Considering plasma p-tau217, the strongest evidence concerned delayed episodic memory. Participants with positive p-tau217 status showed a significant decline in short story delayed recall, while those in the p-tau217 gray zone displayed an intermediate trajectory. This finding is consistent with the biological role of p-tau217 as a marker closely linked to AD pathophysiology and supports its potential prognostic value in the earliest clinical phases of the disease continuum [19, 42]. Episodic memory impairment, particularly on delayed recall tasks, is a core cognitive feature of prodromal and clinical AD [43]. The observation that such changes were detectable in SCD patients with abnormal plasma p-tau217 suggests that subtle AD-related cognitive decline may already be measurable before patients meet criteria for MCI. A particularly relevant aspect of our results is the behavior of participants in the p-tau217 gray zone. These individuals showed memory changes that appeared intermediate between p-tau217 negative and positive patients. This supports the view that gray-zone values should not be considered clinically neutral. Rather, they may identify individuals in a transitional biological state, in whom AD-related pathology is emerging but has not yet reached levels associated with a clearly positive biomarker profile [12, 44]. From a clinical perspective, this observation is important because it suggests that gray-zone p-tau217 values may warrant closer longitudinal monitoring, especially in SCD population [23]. Importantly, p-tau217 positivity was also associated with worsening performance on visual search. This finding extends the cognitive profile associated with plasma p-tau217 beyond episodic memory and suggests early involvement of visual attentional processes. Although memory impairment has traditionally been considered the hallmark of early AD, increasing evidence indicates that attentional functions may also be affected in the preclinical and SCD phases, particularly among individuals with underlying AD pathology [45]. This finding is clinically relevant because attentional measures may contribute to identifying SCD individuals with biological evidence of AD who are already following an unfavorable cognitive trajectory. On the other hand, the findings for category fluency require a more nuanced interpretation. Indeed, we found that p-tau217 negative patients improved over time, whereas p-tau217 positive ones failed to show a comparable positive trajectory. This pattern may reflect the intrinsic heterogeneity of SCD. Indeed, SCD is not only driven by neurodegenerative disease but may be related to potentially reversible or fluctuating factors, including affective symptoms, stress, sleep disturbances, medical comorbidities, or attentional inefficiency [22]. Improvement in p-tau217 negative patients may therefore reflect clinical stabilization, reduction of non-neurodegenerative contributors, or regression toward more efficient cognitive functioning over time [22]. Conversely, the absence of comparable improvement in p-tau217 positive patients may indicate that underlying AD pathology limits cognitive recovery or compensatory mechanisms.

Compared with p-tau217, plasma p-tau181 showed a less robust association with longitudinal cognitive outcomes. Positive p-tau181 status was associated with decline in some memory measures, including short story and RAVLT at post hoc analysis [46]. Nevertheless, the overall pattern was less consistent, and interaction effects were generally weaker. Although previous studies have demonstrated the prognostic value of plasma p-tau181 for detecting cognitive decline, its role appears to be more limited, and overall less prominent, when compared with p-tau217 [46–48]. This is in line with the growing evidence suggesting that p-tau217 may be more sensitive than p-tau181 to early AD-related biological changes. Although both biomarkers reflect tau phosphorylation in the context of amyloid-related pathology, p-tau217 appears to better capture early AD-specific processes and may therefore be more suitable for detecting subtle cognitive vulnerability in preclinical populations such as SCD [49, 50].

The results for NfL were less specific. NfL status was not associated with a clear AD-like longitudinal cognitive profile. Some findings emerged in working memory, and NfL-negative patients showed lower overall performance on forward spatial and backward digit span, but these associations did not define a coherent pattern of progressive decline. This is biologically plausible, given that NfL reflects neuroaxonal injury across a wide range of neurological conditions and is not specific to AD pathology [51]. In SCD populations, elevated NfL may therefore signal general neuronal vulnerability, comorbidity burden, vascular or other neurodegenerative processes, rather than AD-specific cognitive decline. At the same time, variability in NfL levels may also occur among SCD patients with underlying AD, reflecting differences in the biological stage of the disease and in the degree of ongoing neurodegeneration [1]. Thus, some individuals with AD pathology may present with relatively low NfL levels if neuroaxonal injury is still limited, whereas others may show higher NfL concentrations when neurodegeneration is more advanced or more biologically active. The divergence between p-tau217 and NfL in our study may therefore indicate that these biomarkers capture complementary aspects of the disease process: p-tau217 more closely reflecting AD-specific amyloid-related tau pathology, and NfL reflecting the downstream intensity of neuroaxonal damage [52, 53].

Third, although the present analysis focused on plasma p-tau217, p-tau181, and NfL, these biomarkers do not capture the full spectrum of biological mechanisms involved in AD pathophysiology, including inflammatory, astroglial, vascular, and metabolic pathways. Future studies incorporating additional markers, such as GFAP and other indices of neuroinflammation or glial activation, together with longer follow-up periods, will be needed to clarify how these mechanisms contribute to domain-specific cognitive trajectories in SCD. Finally, SCD is clinically heterogeneous, and residual confounding by affective symptoms, sleep quality, vascular burden, or other medical factors cannot be fully excluded.

Several limitations should be acknowledged. First, the sample size was modest, particularly after stratification according to plasma biomarker status. Although the three-tier p-tau217 classification reflected the previously described two-cut-off approach, the resulting subgroup sizes were small, which may have limited statistical power, increased the uncertainty of subgroup estimates, and raised the risk of Type II errors. This limitation applies to all biomarker analyses, although it should be considered particularly when interpreting the less consistent findings observed for p-tau181 and NfL. Second, the follow-up duration of two years may be insufficient to capture the full extent of cognitive decline in SCD, a stage in which progression is often slow and heterogeneous. However, the aim of the present study was not to reconstruct the full natural history of AD, nor to assess clinical conversion to MCI or dementia, but rather to detect short-term changes in domain-specific neuropsychological performance. Therefore, biomarker positivity should not be interpreted as indicating an irreversible point of no return, but rather as a signal of early biological and cognitive vulnerability that may warrant closer longitudinal monitoring. Third, although the present analysis focused on plasma p-tau217, p-tau181, and NfL, these biomarkers do not capture the full spectrum of biological mechanisms involved in AD pathophysiology, including inflammatory, astroglial, vascular, and metabolic pathways. Future studies incorporating additional markers, such as GFAP and other indices of neuroinflammation or glial activation, together with longer follow-up periods, will be needed to clarify how these mechanisms contribute to domain-specific cognitive trajectories in SCD. Finally, SCD is clinically heterogeneous, and residual confounding by affective symptoms, sleep quality, vascular burden, systemic inflammatory status, or other medical factors cannot be fully excluded. Moreover, baseline systemic inflammatory markers which may influence cognitive disturbances were not systematically available and therefore could not be included as covariates. Nevertheless, the present study provides evidence that plasma biomarkers, especially p-tau217, are associated with domain-specific cognitive trajectories in SCD, even in a relatively short follow-up time. By focusing on longitudinal changes across individual cognitive domains rather than on global cognitive measures alone, our findings suggest that p-tau217 positivity may identify a subgroup of patients showing early vulnerability in episodic memory and attentional functioning. This pattern, together with the weaker associations observed for p-tau181 and the less specific profile associated with NfL, suggests that p-tau217 shows more consistent associations with early domain-specific cognitive changes in SCD, supporting its role in biological and cognitive stratification.

In conclusion, plasma p-tau217 may represent a promising tool for identifying SCD individuals more likely to harbor early AD-related pathology and to follow an unfavorable cognitive trajectory. Abnormal p-tau217 levels may help distinguish these patients from those whose subjective complaints are potentially related to non-degenerative or reversible causes. This distinction is particularly relevant in the current therapeutic landscape, where disease-modifying treatments and early biological diagnosis are gaining increasing importance. In this context, plasma biomarkers may offer a scalable and minimally invasive approach to guide closer follow-up, biomarker confirmation, and enrollment in preventive or early-intervention trials.

## Data and materials availability

All study data, including raw and analyzed data, and materials that support the findings of this study are available from the corresponding author (B.N.) upon reasonable request.

## Supplementary Information

Below is the link to the electronic supplementary material.

Supplementary file1 (DOCX 40 KB)
