# Supplementary material for: Plasma p-tau217 best captures early longitudinal cognitive changes in subjective cognitive decline compared with p-tau181 and NfL
Source: J Neurol. 2026 Jun 26;273(7):422. doi: 10.1007/s00415-026-13966-z (PMC13309395; doi:10.1007/s00415-026-13966-z)
Supplement: Supplementary file 1 — Supplementary file1 (DOCX 40 KB) [file 415_2026_13966_MOESM1_ESM.docx]

**Supplementary Table 1. Longitudinal mixed-effects models of neuropsychological outcomes according to plasma p-tau217 status**

|  | **Fixed Effects** | **β** | **SE** | ***p*** |
| --- | --- | --- | --- | --- |
| **MMSE** | Time | 0.502 | 0.412 | 0.230 |
|  | p-tau217 status (GZ) | 0.301 | 0.588 | 0.609 |
|  | p-tau217 status (positive) | -0.923 | 0.655 | 0.163 |
|  | Time x p-tau217 (GZ) | -0.592 | 0.652 | 0.370 |
|  | Time x p-tau217 (positive) | 0.174 | 0.745 | 0.816 |
| **Short Story Immediate Recall** | Time | 0.761 | 1.002 | 0.452 |
|  | p-tau217 status (GZ) | 1.000 | 1.340 | 0.458 |
|  | p-tau217 status (positive) | -1.333 | 1.547 | 0.392 |
|  | Time x p-tau217 (GZ) | -1.904 | 1.584 | 0.236 |
|  | Time x p-tau217 (positive) | -2.984 | 1.830 | 0.111 |
| **Short Story Delayed Recall** | Time | 0.507 | 0.894 | 0.573 |
|  | p-tau217 status (GZ) | -0.166 | 1.342 | 0.901 |
|  | p-tau217 status (positive) | -2.381 | 1.550 | 0.129 |
|  | Time x p-tau217 (GZ) | -2.792 | 1.414 | 0.055 |
|  | Time x p-tau217 (positive) | -4.284 | 1.633 | **0.012** |
| **Rey Auditory Verbal Learning Test Immediate Recall** | Time | -2.716 | 1.853 | 0.151 |
|  | p-tau217 status (GZ) | -0.635 | 3.323 | 0.849 |
|  | p-tau217 status (positive) | -5.090 | 3.787 | 0.184 |
|  | Time x p-tau217 (GZ) | -1.314 | 2.931 | 0.656 |
|  | Time x p-tau217 (positive) | 0.868 | 3.469 | 0.804 |
| **Rey Auditory Verbal Learning Test Delayed Recall** | Time | -0.985 | 0.596 | 0.106 |
|  | p-tau217 status (GZ) | -0.257 | 0.945 | 0.786 |
|  | p-tau217 status (positive) | -1.094 | 1.082 | 0.315 |
|  | Time x p-tau217 (GZ) | -0.215 | 0.943 | 0.820 |
|  | Time x p-tau217 (positive) | -0.567 | 1.111 | 0.612 |
| **Rey-Osterrieth Complex Figure copy** | Time | -0.503 | 0.904 | 0.581 |
|  | p-tau217 status (GZ) | -1.379 | 1.023 | 0.181 |
|  | p-tau217 status (positive) | -2.901 | 1.139 | **0.012** |
|  | Time x p-tau217 (GZ) | -0.292 | 1.436 | 0.839 |
|  | Time x p-tau217 (positive) | 1.510 | 1.609 | 0.353 |
| **Rey-Osterrieth complex figure recall** | Time | 0.495 | 1.103 | 0.656 |
|  | p-tau217 status (GZ) | -3.108 | 1.867 | 0.101 |
|  | p-tau217 status (positive) | -1.669 | 2.079 | 0.425 |
|  | Time x p-tau217 (GZ) | -0.656 | 1.744 | 0.709 |
|  | Time x p-tau217 (positive) | -2.917 | 1.998 | 0.152 |
| **Category Fluency** | Time | 6.020 | 1.745 | **0.001** |
|  | p-tau217 status (GZ) | 0.141 | 3.211 | 0.965 |
|  | p-tau217 status (positive) | -0.217 | 3.571 | 0.951 |
|  | Time x p-tau217 (GZ) | -2.856 | 2.726 | 0.301 |
|  | Time x p-tau217 (positive) | 7.308 | 3.122 | **0.024** |
| **Phonemic Fluency** | Time | 5.064 | 1.941 | **0.012** |
|  | p-tau217 status (GZ) | -0.041 | 4.047 | 0.991 |
|  | p-tau217 status (positive) | -1.893 | 4.506 | 0.676 |
|  | Time x p-tau217 (GZ) | -1.682 | 3.070 | 0.586 |
|  | Time x p-tau217 (positive) | 3.760 | 3.527 | 0.292 |
| **Stroop Test** | Time | 1.647 | 1.318 | 0.218 |
|  | p-tau217 status (GZ) | 0.758 | 3.235 | 0.815 |
|  | p-tau217 status (positive) | 6.535 | 3.600 | 0.075 |
|  | Time x p-tau217 (GZ) | -1.886 | 2.057 | 0.365 |
|  | Time x p-tau217 (positive) | -3.096 | 2.453 | 0.214 |
| **Trail Making Test part A** | Time | 6.476 | 2.942 | **0.033** |
|  | p-tau217 status (GZ) | -0.023 | 5.145 | 0.996 |
|  | p-tau217 status (positive) | 5.729 | 6.252 | 0.362 |
|  | Time x p-tau217 (GZ) | -5.381 | 4.738 | 0.263 |
|  | Time x p-tau217 (positive) | -3.926 | 5.721 | 0.496 |
| **Trail Making test part B** | Time | 22.381 | 6.443 | **0.001** |
|  | p-tau217 status (GZ) | -8.006 | 12.765 | 0.532 |
|  | p-tau217 status (positive) | 14.623 | 14.682 | 0.323 |
|  | Time x p-tau217 (GZ) | -6.946 | 10.382 | 0.507 |
|  | Time x p-tau217 (positive) | -4.137 | 12.574 | 0.743 |
| **Visual Search** | Time | 0.384 | 1.429 | 0.789 |
|  | p-tau217 status (GZ) | -0.419 | 2.174 | 0.848 |
|  | p-tau217 status (positive) | -0.953 | 2.417 | 0.694 |
|  | Time x p-tau217 (GZ) | 0.793 | 2.185 | 0.718 |
|  | Time x p-tau217 (positive) | -4.954 | 2.423 | **0.047** |
| **Frontal Assessment Battery** | Time | 0.331 | 0.412 | 0.426 |
|  | p-tau217 status (GZ) | 0.049 | 0.545 | 0.929 |
|  | p-tau217 status (positive) | -0.758 | 0.607 | 0.215 |
|  | Time x p-tau217 (GZ) | -0.551 | 0.655 | 0.405 |
|  | Time x p-tau217 (positive) | -0.853 | 0.718 | 0.242 |
| **Naming** | Time | -0.229 | 0.224 | 0.316 |
|  | p-tau217 status (GZ) | -0.306 | 0.281 | 0.279 |
|  | p-tau217 status (positive) | -0.201 | 0.313 | 0.522 |
|  | Time x p-tau217 (GZ) | 0.309 | 0.363 | 0.401 |
|  | Time x p-tau217 (positive) | -0.137 | 0.404 | 0.736 |
| **Verbal Span Forward** | Time | -0.125 | 0.246 | 0.614 |
|  | p-tau217 status (GZ) | 0.620 | 0.337 | 0.069 |
|  | p-tau217 status (positive) | -0.192 | 0.389 | 0.623 |
|  | Time x p-tau217 (GZ) | -0.029 | 0.389 | 0.941 |
|  | Time x p-tau217 (positive) | 0.459 | 0.463 | 0.326 |
| **Verbal Span Backward** | Time | 0.360 | 0.247 | 0.154 |
|  | p-tau217 status (GZ) | 0.396 | 0.383 | 0.305 |
|  | p-tau217 status (positive) | -0.025 | 0.442 | 0.954 |
|  | Time x p-tau217 (GZ) | 0.350 | 0.391 | 0.376 |
|  | Time x p-tau217 (positive) | -0.041 | 0.466 | 0.931 |
| **Spatial Span Forward** | Time | -0.348 | 0.219 | 0.120 |
|  | p-tau217 status (GZ) | 0.015 | 0.282 | 0.957 |
|  | p-tau217 status (positive) | -0.564 | 0.319 | 0.081 |
|  | Time x p-tau217 (GZ) | -0.133 | 0.352 | 0.707 |
|  | Time x p-tau217 (positive) | 0.178 | 0.401 | 0.659 |
| **Spatial Span Backward** | Time | 0.133 | 0.252 | 0.598 |
|  | p-tau217 status (GZ) | 0.245 | 0.338 | 0.472 |
|  | p-tau217 status (positive) | -0.088 | 0.383 | 0.818 |
|  | Time x p-tau217 (GZ) | -0.399 | 0.404 | 0.329 |
|  | Time x p-tau217 (positive) | -0.520 | 0.460 | 0.265 |

Values are fixed-effect estimates from linear mixed-effects models with random intercepts for participants. Each model included Time, plasma p-tau217 status, and the Time × p-tau217 interaction as predictors. The negative group was used as the reference category. Coefficients for p-tau217 gray zone and positive status represent differences relative to the negative group at baseline. Interaction terms represent differential longitudinal change over time relative to the negative group. Positive β values indicate increasing scores over time or higher performance relative to the reference group, whereas negative β values indicate decreasing scores or lower performance, except for timed tests (e.g., Trail Making Test, Stroop). Abbreviations: β, regression coefficient; SE, standard error; SCD, subjective cognitive decline. Statistically significant results are highlighted in bold. Statistical significance are reported in **bold** character.

**Supplementary Table 2. Longitudinal mixed-effects models of neuropsychological outcomes according to plasma p-tau181 status**

|  | **Fixed Effects** | **β** | **SE** | ***p*** |
| --- | --- | --- | --- | --- |
| **MMSE** | Time | 0.760 | 0.378 | 0.051 |
|  | p-tau181 status (positive) | 0.277 | 0.520 | 0.595 |
|  | Time x p-tau181 (positive) | -1.043 | 0.580 | 0.079 |
| **Short Story Immediate Recall** | Time | -0.307 | 0.860 | 0.722 |
|  | p-tau181 status (positive) | -1.119 | 1.280 | 0.385 |
|  | Time x p-tau181 (positive) | -1.192 | 1.344 | 0.380 |
| **Short Story Delayed Recall** | Time | -1.027 | 0.819 | 0.216 |
|  | p-tau181 status (positive) | -2.307 | 1.305 | 0.081 |
|  | Time x p-tau181 (positive) | -0.972 | 1.281 | 0.452 |
| **Rey Auditory Verbal Learning Test Immediate Recall** | Time | -2.381 | 1.615 | 0.148 |
|  | p-tau181 status (positive) | -1.147 | 2.865 | 0.690 |
|  | Time x p-tau181 (positive) | -2.658 | 2.510 | 0.296 |
| **Rey Auditory Verbal Learning Test Delayed Recall** | Time | -1.067 | 0.504 | **0.040** |
|  | p-tau181 status (positive) | -0.435 | 0.798 | 0.587 |
|  | Time x p-tau181 (positive) | -0.564 | 0.783 | 0.475 |
| **Rey-Osterrieth Complex Figure copy** | Time | -0.474 | 0.863 | 0.586 |
|  | p-tau181 status (positive) | -1.241 | 1.002 | 0.219 |
|  | Time x p-tau181 (positive) | -0.871 | 1.335 | 0.517 |
| **Rey-Osterrieth complex figure recall** | Time | -0.176 | 1.036 | 0.865 |
|  | p-tau181 status (positive) | -1.046 | 1.724 | 0.546 |
|  | Time x p-tau181 (positive) | -1.421 | 1.589 | 0.376 |
| **Category Fluency** | Time | 4.900 | 1.563 | **0.003** |
|  | p-tau181 status (positive) | 0.883 | 2.898 | 0.761 |
|  | Time x p-tau181 (positive) | -2.514 | 2.407 | 0.302 |
| **Phonemic Fluency** | Time | 4.593 | 1.784 | **0.013** |
|  | p-tau181 status (positive) | -1.124 | 3.379 | 0.740 |
|  | Time x p-tau181 (positive) | -2.632 | 2.739 | 0.341 |
| **Stroop Test** | Time | -0.269 | 1.805 | 0.882 |
|  | p-tau181 status (positive) | 1.222 | 3.090 | 0.694 |
|  | Time x p-tau181 (positive) | 4.232 | 2.883 | 0.150 |
| **Trail Making Test part A** | Time | 5.488 | 2.938 | 0.068 |
|  | p-tau181 status (positive) | 4.126 | 4.423 | 0.354 |
|  | Time x p-tau181 (positive) | -1.808 | 4.571 | 0.694 |
| **Trail Making test part B** | Time | 22.381 | 6.443 | **0.001** |
|  | p-tau181 status (positive) | 24.062 | 11.255 | **0.036** |
|  | Time x p-tau181 (positive) | -7.870 | 10.719 | 0.467 |
| **Visual Search** | Time | 0.294 | 1.477 | 0.842 |
|  | p-tau181 status (positive) | -1.473 | 2.077 | 0.480 |
|  | Time x p-tau181 (positive) | -3.859 | 2.228 | 0.090 |
| **Frontal Assessment Battery** | Time | 0.180 | 0.419 | 0.669 |
|  | p-tau181 status (positive) | -0.440 | 0.486 | 0.368 |
|  | Time x p-tau181 (positive) | -0.724 | 0.634 | 0.259 |
| **Naming** | Time | -0.051 | 0.201 | 0.803 |
|  | p-tau181 status (positive) | -0.174 | 0.228 | 0.449 |
|  | Time x p-tau181 (positive) | -0.291 | 0.305 | 0.349 |
| **Verbal Span Forward** | Time | -0.235 | 0.217 | 0.285 |
|  | p-tau181 status (positive) | -0.236 | 0.303 | 0.439 |
|  | Time x p-tau181 (positive) | 0.145 | 0.341 | 0.673 |
| **Verbal Span Backward** | Time | -0.384 | 0.218 | 0.085 |
|  | p-tau181 status (positive) | -0.468 | 0.312 | 0.138 |
|  | Time x p-tau181 (positive) | 0.470 | 0.343 | 0.178 |
| **Spatial Span Forward** | Time | -0.205 | 0.195 | 0.297 |
|  | p-tau181 status (positive) | 0.134 | 0.267 | 0.618 |
|  | Time x p-tau181 (positive) | -0.220 | 0.300 | 0.467 |
| **Spatial Span Backward** | Time | -0.013 | 0.224 | 0.953 |
|  | p-tau181 status (positive) | 0.103 | 0.295 | 0.727 |
|  | Time x p-tau181 (positive) | -0.327 | 0.346 | 0.349 |

Values are fixed-effect estimates from linear mixed-effects models with random intercepts for participants. Each model included Time, plasma p-tau181 status, and the Time × p-tau181 interaction as predictors. The negative group was used as the reference category. Coefficients for p-tau181 positive status represent differences relative to the negative group at baseline. Interaction terms represent differential longitudinal change over time relative to the negative group. Positive β values indicate increasing scores over time or higher performance relative to the reference group, whereas negative β values indicate decreasing scores or lower performance, except for timed tests (e.g., Trail Making Test, Stroop). Abbreviations: β, regression coefficient; SE, standard error; SCD, subjective cognitive decline. Statistically significant results are highlighted in bold. Statistical significance are reported in **bold** character.

**Supplementary Table 3. Longitudinal mixed-effects models of neuropsychological outcomes according to plasma NfL status**

|  | **Fixed Effects** | **β** | **SE** | ***p*** |
| --- | --- | --- | --- | --- |
| **MMSE** | Time | 0.645 | 0.297 | **0.034** |
|  | NfL status (positive) | -0.547 | 0.537 | 0.311 |
|  | Time x NfL (positive) | -0.854 | 0.607 | 0.165 |
| **Short Story Immediate Recall** | Time | 0.024 | 0.739 | 0.974 |
|  | NfL status (positive) | -0.613 | 1.384 | 0.658 |
|  | Time x NfL (positive) | -1.274 | 1.553 | 0.416 |
| **Short Story Delayed Recall** | Time | -0.481 | 0.672 | 0.478 |
|  | NfL status (positive) | -0.609 | 1.477 | 0.681 |
|  | Time x NfL (positive) | -2.685 | 1.413 | 0.063 |
| **Rey Auditory Verbal Learning Test Immediate Recall** | Time | -2.817 | 1.333 | **0.039** |
|  | NfL status (positive) | -1.576 | 3.057 | 0.607 |
|  | Time x NfL (positive) | -1.323 | 2.789 | 0.637 |
| **Rey Auditory Verbal Learning Test Delayed Recall** | Time | -1.024 | 0.432 | **0.021** |
|  | NfL status (positive) | -0.668 | 0.874 | 0.447 |
|  | Time x NfL (positive) | -0.334 | 0.903 | 0.712 |
| **Rey-Osterrieth Complex Figure copy** | Time | -0.379 | 0.642 | 0.557 |
|  | NfL status (positive) | -1.283 | 1.028 | 0.215 |
|  | Time x NfL (positive) | -0.185 | 1.332 | 0.890 |
| **Rey-Osterrieth complex figure recall** | Time | 0.001 | 0.775 | 0.999 |
|  | NfL status (positive) | -1.087 | 1.768 | 0.541 |
|  | Time x NfL (positive) | -0.312 | 1.584 | 0.845 |
| **Category Fluency** | Time | 3.183 | 1.390 | **0.026** |
|  | NfL status (positive) | -4.253 | 3.017 | 0.162 |
|  | Time x NfL (positive) | -0.931 | 2.887 | 0.748 |
| **Phonemic Fluency** | Time | 3.943 | 1.291 | **0.003** |
|  | NfL status (positive) | -0.558 | 3.605 | 0.877 |
|  | Time x NfL (positive) | -1.702 | 2.634 | 0.521 |
| **Stroop Test** | Time | 0.6269 | 1.296 | 0.631 |
|  | NfL status (positive) | 2.935 | 3.089 | 0.345 |
|  | Time x NfL (positive) | 1.974 | 2.671 | 0.463 |
| **Trail Making Test part A** | Time | 5.481 | 2.480 | **0.031** |
|  | NfL status (positive) | 6.503 | 5.453 | 0.236 |
|  | Time x NfL (positive) | -2.848 | 5.288 | 0.592 |
| **Trail Making test part B** | Time | 19.006 | 5.098 | **0.001** |
|  | NfL status (positive) | 14.619 | 12.367 | 0.240 |
|  | Time x NfL (positive) | -11.618 | 11.200 | 0.304 |
| **Visual Search** | Time | 0.450 | 1.109 | 0.686 |
|  | NfL status (positive) | -1.699 | 2.255 | 0.453 |
|  | Time x NfL (positive) | -3.850 | 2.234 | 0.090 |
| **Frontal Assessment Battery** | Time | 0.035 | 0.315 | 0.912 |
|  | NfL status (positive) | -0.534 | 0.509 | 0.297 |
|  | Time x NfL (positive) | -0.328 | 0.634 | 0.607 |
| **Naming** | Time | -0.153 | 0.147 | 0.304 |
|  | NfL status (positive) | -0.200 | 0.236 | 0.399 |
|  | Time x NfL (positive) | 0.045 | 0.302 | 0.881 |
| **Verbal Span Forward** | Time | -0.266 | 0.164 | 0.111 |
|  | NfL status (positive) | -0.375 | 0.316 | 0.240 |
|  | Time x NfL (positive) | 0.714 | 0.340 | **0.040** |
| **Verbal Span Backward** | Time | -0.380 | 0.166 | **0.026** |
|  | NfL status (positive) | -0.575 | 0.358 | 0.113 |
|  | Time x NfL (positive) | 0.818 | 0.345 | **0.022** |
| **Spatial Span Forward** | Time | -0.430 | 0.147 | **0.005** |
|  | NfL status (positive) | -0.594 | 0.276 | **0.034** |
|  | Time x NfL (positive) | 0.301 | 0.305 | 0.328 |
| **Spatial Span Backward** | Time | -0.154 | 0.177 | 0.388 |
|  | NfL status (positive) | -0.343 | 0.307 | 0.268 |
|  | Time x NfL (positive) | 0.141 | 0.367 | 0.703 |

Values are fixed-effect estimates from linear mixed-effects models with random intercepts for participants. Each model included Time, plasma NfL status, and the Time × NfL interaction as predictors. The negative group was used as the reference category. Coefficients for NfL positive status represent differences relative to the negative group at baseline. Interaction terms represent differential longitudinal change over time relative to the negative group. Positive β values indicate increasing scores over time or higher performance relative to the reference group, whereas negative β values indicate decreasing scores or lower performance, except for timed tests (e.g., Trail Making Test, Stroop). Abbreviations: β, regression coefficient; SE, standard error; SCD, subjective cognitive decline. Statistically significant results are highlighted in bold. Statistical significance are reported in **bold** character.
